# Worth Attention

**Published:** 1885-05

**Authors:** 


### WORTH ATTENTION.

Will the readers of this number carefully and heedfully go over the six propositions laid down by Dr. Miller on page 228 as fully demonstrated, and then take plenty of time for their mental digestion, so that they may be entirely comprehended. It will save very much of misapprehension and misunderstanding.

